# Practical Notes on Diagnosis and Treatment

**Published:** 1910-07-16

**Authors:** 


### Practical Notes on Diagnosis and Treatment.

### Atonic Dyspepsia.

The subjects of this form of dyspepsia are mostly debilitated persons, many of whom are anæmic. For the treatment, acids are indicated at the commencement, and a combination of dilute hydrochloric acid, carbolic acid, strychnine, and ginger ments the requirements of many cases.—*Dr. Guthrie Rankin.*

### Tunica Vaginalis.

This is the only sensitive serous membrane and the only one to which a branch of a cerebro-spinal nerve has been traced. In renal colic the tunica vaginalis is hyperæsthetic, but there is no hyperæsthetic area on the skin of the scrotum corresponding to the painful areas connected with other examples of referred visceral pain.

### Suppuration.

In many cases it has been found that abscesses may be completely dispersed by the following method, which has been very successful for abscesses in the neck. The skin having been scrubbed with a boiled nail-brush, several layers of gauze soaked in ether are placed upon the prepared area and the compress covered with oiled silk and bandage. Frequently a corner of the dressing is raised and enough ether poured on to moisten the gauze. In a few days cure may be looked for almost with certainty.

### Dermatology.

The effect of the mental attitude of the patient on the healing of a generalised skin affection is no fetish but a great reality.—*Dr. J. M. H. Macleod.*

### Rudimentary Tabes.

The recognition of early cases of tabes has more than a scientific interest, especially with regard to the prevention of ataxia and of bladder trouble. Ataxia can be delayed by the avoidance of physical over-exertion in the lower limbs, whilst bladder troubles may also be prevented by instructing the patient to empty the bladder every two hours during the day.—*Dr. Purves Stewart.*

### Synovitis of the Knee.

The apparent simplicity of the diagnosis of traumatic synovitis is continually producing a rich crop of errors. Careful inquiry is necessary into the definiteness of the history of trauma and into the question of previous trouble in other joints. The exclusion of gonorrhœal infection should be most carefully gone into, and the possibilities of acute suppurative arthritis and tuberculosis must always be borne in mind. The manipulative examinations of the joint, and especially the "feel" of the capsule give most reliable information. It may be noted that tenderness over the internal semilunar cartilage is usually present in cases of arthritis, but does not mean injury to the cartilage.

